# Effect of Different Carbon Sources on Bacterial Nanocellulose Production and Structure Using the Low pH Resistant Strain *Komagataeibacter Medellinensis*

**DOI:** 10.3390/ma10060639

**Published:** 2017-06-11

**Authors:** Carlos Molina-Ramírez, Margarita Castro, Marlon Osorio, Mabel Torres-Taborda, Beatriz Gómez, Robin Zuluaga, Catalina Gómez, Piedad Gañán, Orlando J. Rojas, Cristina Castro

**Affiliations:** 1 Facultad de Ingeniería Química, Universidad Pontificia Bolivariana, Circular 1°, No. 70-01, Medellín 050031, Colombia; margarita.castro@upb.edu.co (M.C.); marlonandres.osorio@upb.edu.co (M.O.); mabel.torres@upb.edu.co (M.T.-T.); Beatriz.gomez@upb.edu.co (B.G.); catalina.gomezh@upb.edu.co (C.G.); piedad.ganan@upb.edu.co (P.G.); 2 Facultad de Ingeniería Agroindustrial, Universidad Pontificia Bolivariana, Circular 1°, No. 70-01, Medellín 050031, Colombia; robin.zuluaga@upb.edu.co; 3 Biobased Colloids and Materials Group (BiCMat), Department of Bioproducts and Biosystems, School of Chemical Engineering, Aalto University, Espoo 02150, Finland; orlando.rojas@aalto.fi; 4 Facultad de Ingeniería Textil, Universidad Pontificia Bolivariana, Circular 1°, No. 70-01, Medellín 050031, Colombia; cristina.castro@upb.edu.co

**Keywords:** bacterial nanocellulose, carbon source, cellulose crystallization, *Komagataeibacter medellinensis*, static fermentation

## Abstract

Bacterial cellulose (BC) is a polymer obtained by fermentation with microorganism of different genera. Recently, new producer species have been discovered, which require identification of the most important variables affecting cellulose production. In this work, the influence of different carbon sources in BC production by a novel low pH-resistant strain *Komagataeibacter medellinensis* was established. The Hestrin-Schramm culture medium was used as a reference and was compared to other media comprising glucose, fructose, and sucrose, used as carbon sources at three concentrations (1, 2, and 3% *w*/*v*). The BC yield and dynamics of carbon consumption were determined at given fermentation times during cellulose production. While the carbon source did not influence the BC structural characteristics, different production levels were determined: glucose > sucrose > fructose. These results highlight considerations to improve BC industrial production and to establish the BC property space for applications in different fields.

## 1. Introduction

Cellulose is the most abundant biopolymer on Earth, which is produced (10^11^ tons per year) by plants, alga, fungi, and bacteria [1]. Cellulose of plant origin is the most available and used: it is typically extracted by chemical and mechanical treatments from the natural cell wall matrix (comprising by cellulose, lignin, hemicellulose, and waxes) [2]. These treatments produce fibers with high size dispersion and may load the environment with residual components, if not utilized [3].

Gram-negative bacteria in *Komagataeibacter* (former *Gluconacetobacter* and *Acetobacter*) genus are mainly cellulose producer [4]; these bacteria are strictly aerobic and generate bacterial cellulose (BC) as an extracellular product at the air-medium interface at pH between 3 and 7 and temperatures between 28 and 30 °C [5,6,7]. Currently, some bacteria belonging to the *Komagataeibacter* genus have been reported to possess different metabolic preferences and to produce cellulose with different structural characteristics [8]. In fact, differences in the BC structural characteristics can be observed for those produced by different microorganisms using the same carbon source; for example, BC produced by *Acetobacter sp.V6* [9] in glycerol has higher crystallinity than that produced in glucose. This is in contrast to *Acetobacter xilinum* which produce BC with lower crystallinity in glycerol [10].

In the specific case of *K. medellinensis*, it is important to highlight the advantage that the low pH required for BC production reduces the risk for contamination by other microorganisms [11] and, therefore, BC production is not inhibited, as is otherwise the case for other bacteria of the same *Komagataeibacter* genus [12,13]. Additionally, in low pH conditions *K. medellinensis* produces cellulose that offers alternatives to modify it by alteration on culture media, this kind of in situ modifications of cellulose materials have a promising industrial application to paints, coatings, composite materials, or even biomedical devices [14,15].

The most studied species for cellulose production is *Komagataeibacter xylinus* (former *Gluconacetobacter xylinus* [16]. However, novel cellulose producer strains have been considered, such as *K. medellinensis*, which is Gram-negative bacilli, with 1–3 μm in length and 0.6 to 0.7 μm in width and are presented individually, in pairs or chains; also they are negative oxidase and positive catalase [17]. The colonies formed by this organism are beige, round, rough, and opaque.

The culture media commonly used for BC production require mainly a carbon and nitrogen source and salts to buffer the pH [18,19,20]. However, the optimum conditions for cellulose production with this microorganism have not been established yet. In fact, It is well known that there may be differences in metabolic preferences for microorganisms in the same genus between different species [21]. For example, *K. xylinus* have been tested for cellulose production on different carbon sources and the best results were determined when sucrose, glucose, fructose, or mannitol were used; fructose was reported to produce a small yield while glucose was considered the main carbon source for BC production compared to the other three sugars [8]. Compared to fermentation with glucose, fructose has been reported to produce low yields for the first four days while the highest production occurred on the fifth day [10,18,19,20,22,23,24,25,26]. This is because sucrose is a disaccharide composed of two hexoses (glucose and fructose) that must be hydrolyzed before its use in cellulose synthesis.

Fermentation process for cellulose production can be made under static or stirred conditions [27,28]. The static method is the most used to obtain BC in the laboratory, where the polymer grows into pellicles or films; meanwhile the agitated method is the most studied at industrial and biotechnological levels because it produces a higher level of oxygen transfer, which favors the production of cellulose. However, the agitated medium can induce cellular mutability such that a loss of BC productive capacity an occur and the BC produced may have low mechanical strength, crystallinity, and degree of polymerization [27,22,29,30,31].

In this work, we inquired into the effect of carbon source on BC production yield and cellulose structure by using the novel acid-resistant strain *K. medellinensis*, as well as the dynamic of growth. The results will facilitate future work on how to improve BC production and the methods to tailor BC properties for different applications.

## 2. Results

### 2.1. Effect of Carbon Source on Bacterial Cellulose Production

**Figure 1:**
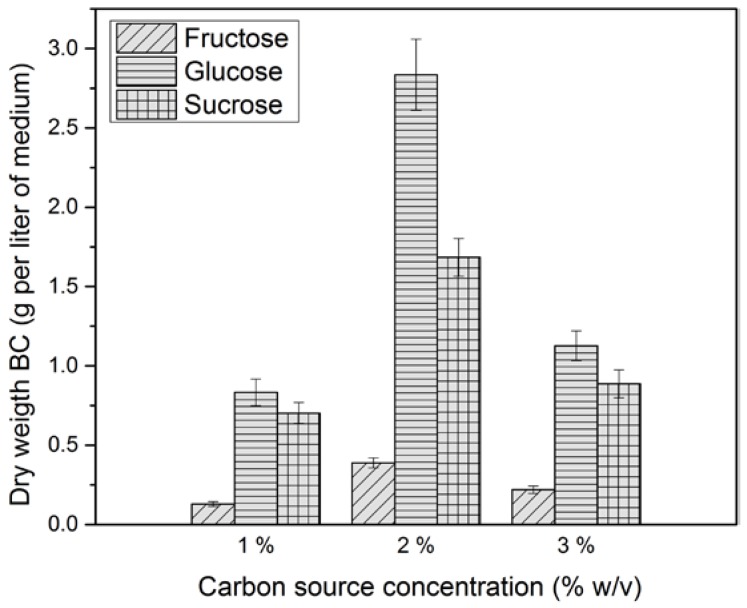
Bacterial cellulose yield in each carbon source culture medium at 1, 2, 3% *w*/*v* at the eighth day of fermentation.

**Figure 2:**
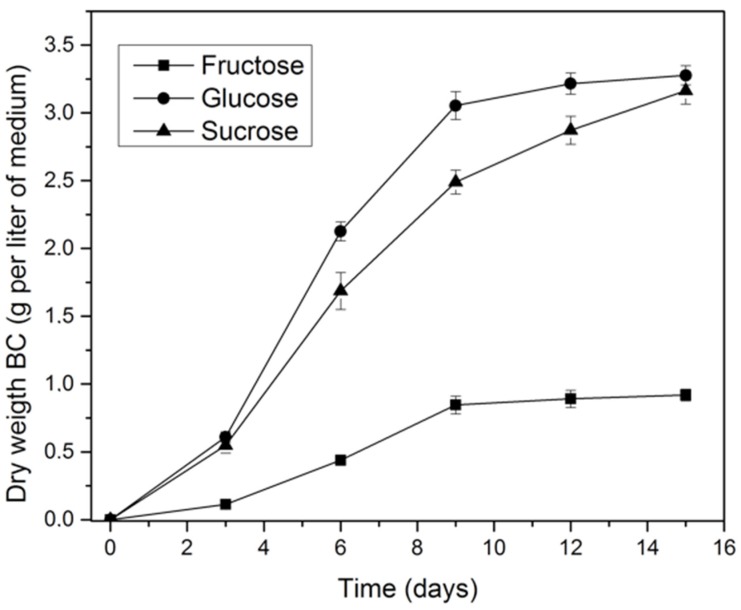
Bacterial cellulose produced by *K. medellinesis* in each culture medium with a substrate of 2%.

The effect of carbon source concentration (1, 2, 3% *w*/*v*) on bacterial cellulose production using the native strain *K. medellinensis* was evaluated (Figure 1). The highest BC yield on the basis of dry weight of the membranes per volume of medium was obtained at 2% *w*/*v* carbon source concentration, for all sources. The BC yield was 2.80, 0.38, and 1.68 g/L using a 2% *w*/*v* of glucose, fructose, and sucrose, respectively. Compared with the dry weight of the membranes obtained from fructose and sucrose, a clear difference was observed for BC production from glucose. The results are in agreement with the observations of Castro et al. [32], Embuscado et al. [33], and others who have indicated that BC production is affected by carbon source type and concentration. The dynamics of BC production for the three different carbon sources evaluated at 2% *w*/*v* during 15 days is shown in Figure 2.

For *K. medellinensis*, the highest yield of cellulose production (3.3 g/L) after 15 days was obtained in the glucose culture medium used as a carbon source, which has been observed and analyzed by [25], [34] for *G. xylinus* [16]. These researchers reported that glucose acted not only as an energy source, but also as a perfect precursor for cellulose polymerization; consequently, glucose is commonly used as a source for cellulose production by strains of this genus.

**Figure 3:**
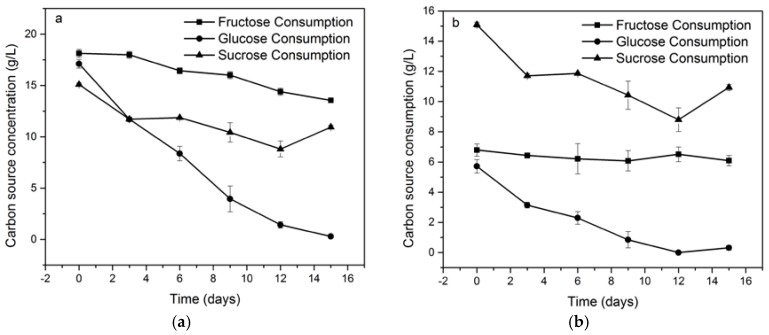
The dynamics of carbon source consumption: Panel (**a**) shows the dynamics of consumption for glucose, fructose and sucrose, each as whole and unique carbon source; panel (**b**) shows a magnified view of the dynamics of consumption of sucrose and its monomeric components, glucose, and fructose. Note that only glucose is consumed after sucrose hydrolysis and the fructose concentration remains the same.

In order to evaluate the consumption rate for each carbon source at 2% *w*/*v*, the carbon source concentration was measured during fermentation for 15 days, as shown in Figure 3a. The product-substrate yield (Y_PS_) during the same time was calculated and the results are shown in Table 1. In the sucrose medium, the glucose and fructose content was also determined in order to measure the actual carbon source consumption (Figure 3b), this is because sucrose is a dimer comprised of glucose and fructose and it is expected that microorganisms break sucrose to metabolize it.

### 2.2. Effect of Carbon Source on K. medellinensis Growth

**Figure 4:**
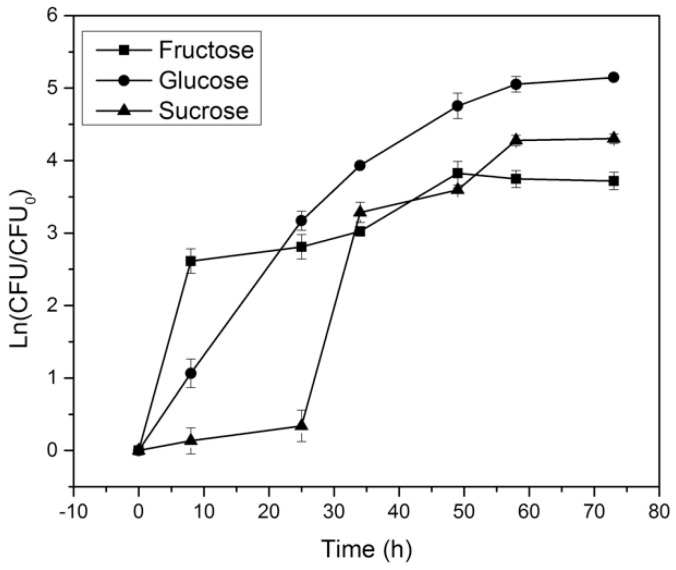
Growth curve of *K. medellinensis* with glucose, fructose and sucrose at 2% *w*/*v*.

The effect on microorganism growth of glucose, fructose, and sucrose (2% *w*/*v*) used as a carbon source in the culture media is shown in Figure 4. For glucose and fructose, the microorganism began to grow without a clear lag phase, indicating that the microorganism did not require a phase adjustment for replication in these carbon sources. In contrast, the sucrose medium displayed a lag phase of 25 h. Therefore, the affinity by *K. medellinesis* for glucose is greater than that for fructose and sucrose.

### 2.3. BC Film Characterization

#### 2.3.1. Scanning Electronic Microscopy (SEM)

**Figure 5:**
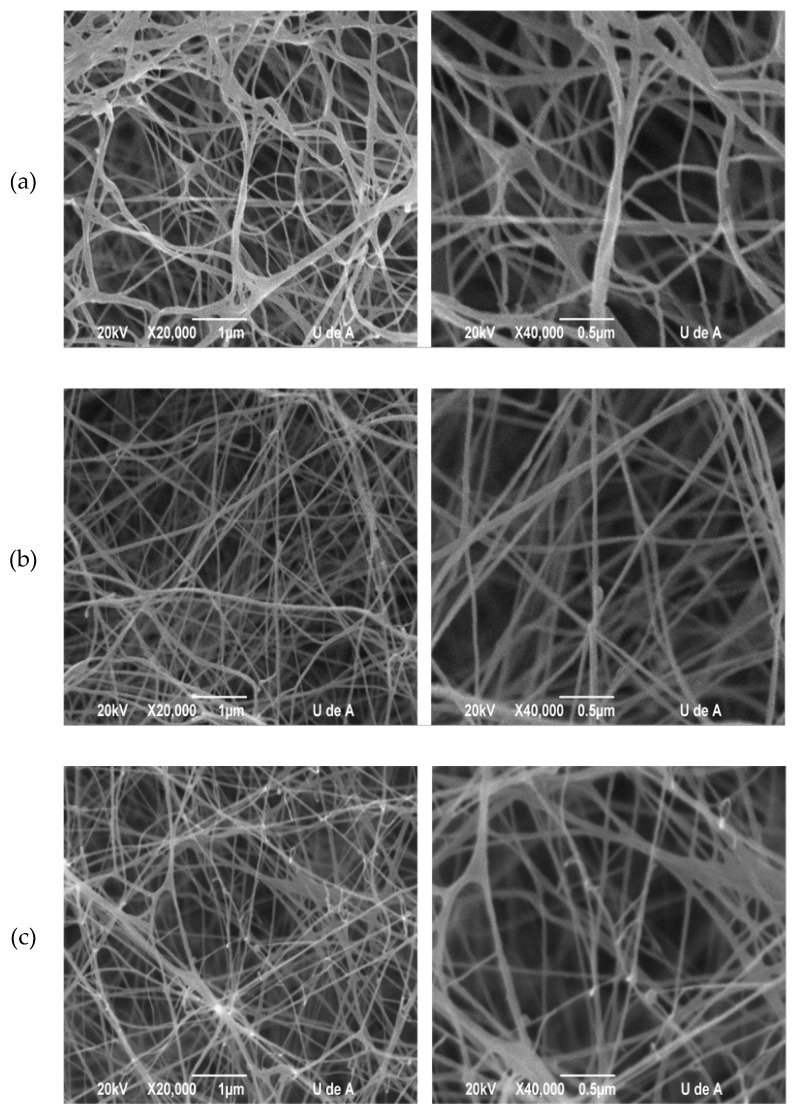
SEM micrograph of bacterial cellulose network obtained from different media: (**a**) Fructose; (**b**) Glucose; and (**c**) Sucrose. The left panel shows the 3D cellulose network at 20,000× and the right panel shows the 3D cellulose network at 40,000×.

The morphology characteristics of BC membranes produced by *K. medellinensis* from the different culture media was evaluated by scanning electronic microscopy (SEM), Figure 5. In all cases the membranes comprised a network of nanoribbons; however, differences in porosity were observed. In Figure 5a a less dense network of nanoribbons is observed from glucose, compared to the cases of fructose and sucrose, Figure 5b,c, respectively. The porosity was determined from the SEM images by using ImageJ software (NIMH, Bethesda, MD, USA) and the results are shown in Table 2. BC membrane from fructose showed to be lesser porous than other membranes from glucose and sucrose.

#### 2.3.2. Attenuated Total Reflection Fourier Transformed Infrared Spectroscopy (ATR-FTIR)

**Figure 6:**
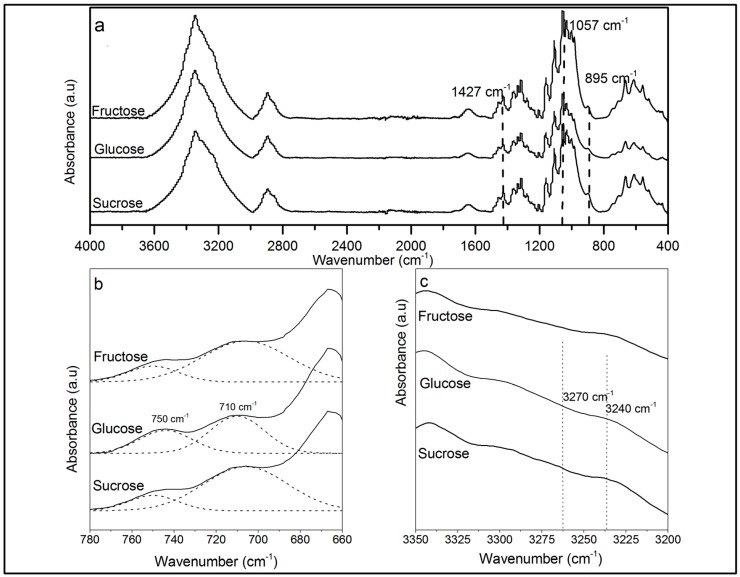
ATR-FT-IR spectra of bacterial cellulose produced in each media: (**a**) complete spectra; (**b**) characteristic bands at 710 cm^−1^ and 750 cm^−1^; and (**c**) characteristics band at 3240 cm^−1^ and 3270 cm^−1^. Straight line: fragment original spectra; dotted line: deconvolution.

The FTIR spectra of cellulose samples prepared from different media are shown in Figure 6a. The spectra exhibited the same vibration bands, indicating that BC produced from the three different media had the same chemical structure. All of the spectra revealed the characteristic bands of the cellulose I crystal structure by the existence of vibrations at 1057 cm^−1^ and 1427 cm^−1^ assigned to C–O stretching and CH_2_ bending, respectively, as well as the 895 cm^−1^ band assigned to β-linkage of cellulose [35,36]. Other characteristics bands for I_α_ and I_β_ allomorphs are shown in Figure 6b,c; these bands are 3240 and 750 cm^−1^ for the I_α_ allomorph and 3270 and 710 cm^−1^ for the I_β_ allomorph, demonstrating the capacity of *K. medellinensis* to produce both allomorphs at the same time. The I_α_ and I_β_ fractions in the BC produced from three media were determined by the Fourier deconvolution with OMNIC software of 710 and 750 cm^−1^ bands, and the results are shown in Table 3. The I_α_ fraction of BC produced was similar for all substrates and higher compared to the I_β_ fraction, indicating that the carbon source had no effect on the crystal conformation. The predominance of the Iα allomorph is in agreement to other findings for bacterial cellulose [37].

#### 2.3.3. X-ray Diffraction

**Figure 7:**
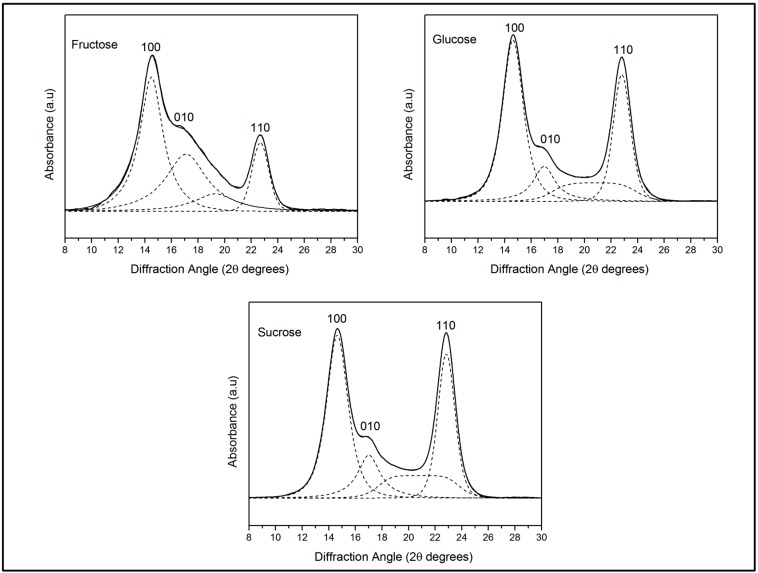
XDR patterns of bacterial cellulose produced from different substrate: (**a**) glucose; (**b**) fructose; and (**c**) sucrose.

The crystalline characteristics of BC produced with the three different media were evaluated using XRD patterns, Figure 7. For the three BC membranes, three resolved peaks assigned to 100, 010, and 110 crystallographic planes were apparent, corresponding to diffraction angles of 14.4°, 16.7°, and 22.7°, respectively, and indexed according to the cellulose Iα indexation described by Sugiyama, et al. [37]. The peaks were deconvoluted using the Voight peak function to calculate the crystallinity index, the interplanar crystal distance (d-spacing) and apparent crystallite size (ACS), Table 4.

## 3. Discussion

Although glucose and fructose have similar chemical structures and the microorganism uses the same metabolic pathway to produce cellulose from these sources, compared to fructose (2% *w*/*v*) the yield with glucose was nearly 86% higher. This highlights a clear substrate inhibition by fructose. A possible explanation for this observation is that the microorganism must use an isomerase to convert fructose into glucose before the polymerization begins; therefore, the microorganism requires greater energy due to enzyme synthesis, yielding a lower BC production, as suggested by other authors [8,38,39,40]. Another possible explanation is that the microorganism grows initially on glucose (HS medium) before its uses in a culture medium with fructose, which provoked a negative effect on enzymatic activity of fructokinase (FK) and so the possibility to phosphorylate the fructose to incorporate it into the metabolism, as was demonstrated by [41] for *G. xilynus* [16].

In Figure 2 it was observed that the BC production started at the third day in all culture media; however, the weight of the membranes obtained in glucose was higher compared to those obtained using fructose and sucrose for the first nine fermentation days. After this time, the mass of the membranes produced in glucose was almost constant, while that for sucrose maintained an increasing trend.

This behavior could be explained by the possibility that fructose remained in the culture medium with sucrose as carbon source. It is probable that fructose produced by sucrose hydrolysis acted as an osmotic stressor since its concentration did not vary during the 15 days of fermentation as is shown in Figure 3. The presence of solutes that cannot diffuse across the cell membrane (i.e., sugars) could trigger defense mechanisms against osmotic stress [42] and one of them is exopolysaccharide (EPS) production to protect itself. This effect was observed by Seesuriyachan et al. for other exopolysaccharides [43]. Therefore, in the medium with sucrose, the lower concentration of glucose, compared to the medium that contained glucose as the sole carbon source, instead of producing biomass, a larger part of glucose was used to produce BC, as shown in Table 1.

Furthermore, in Figure 1 it is observed that the cellulose yield was higher in glucose than sucrose. According to some authors, this is because the microorganism needs to hydrolyze the sucrose into glucose and fructose to incorporate the glucose into its metabolism [18], but owing to the fact that the complete genome of *K. medellinensis* is sequenced [44] and, thus, the metabolic pathways known, the sucrose hydrolysis by this strain was not possible [45]. Therefore, the sucrose hydrolysis was caused by acid hydrolysis due to citric acid being added to the medium and the high temperature during the sterilization process, as can be confirmed in Figure 3.

The less porous BC membrane obtained from the fructose medium than the other two culture media could be related to larger nanoribbon network density from the fructose medium compared to glucose and sucrose media because of the branching rate of the nanoribbon network [46]. In contrast, the BC network from glucose showed the highest porosity and, in turn, a lower visual density of the nanoribbon network in Figure 5 follow by sucrose.

The crystallinity parameters ACS and d-spacing remained unchanged in BC membranes produced from the three different media. The detailed composition of the culture media did not induce differences in the crystallization process of cellulose. However, the abundance of crystallites relative to amorphous zones, determined by the crystallinity index (CI), was slightly higher for BC from fructose compared to glucose and sucrose, which could be related with the length of cellulose chains that constitute the BC ribbons. Probably, ribbons synthesized in fructose were longer than those in the other films obtained from the glucose and sucrose [47] and, therefore, less porous structures resulted, as is shown in Figure 5.

## 4. Materials and Methods 

### 4.1. Culture Media and Growth Conditions

The *Komagataeibacter medellinensis* [17] Yamada (2014) [4] strain used was isolated from homemade vinegar purchased from a local wholesale marketplace. The effect of glucose, fructose, and sucrose as the major carbon sources in cellulose production [32] and its dynamics of growth was evaluated through three different culture media prepared by modifying of standard Hestrin-Schramm medium: carbon source, 2% *w*/*v*; yeast extract, 0.5% *w*/*v*; peptone, 0.5% *w*/*v*; Na_2_HPO_4_, 0.267% *w*/*v* and citric acid, until pH 3.6. The fermentations were performed in liquid culture media under static conditions using vessels with 48 cm^2^ in area and 60 cm^3^ in volume. The culture media were prepared at 1, 2, and 3% *w*/*v* of glucose, fructose, and sucrose (purchased from Honeywell, Merck, and Panreac, respectively), sterilized and inoculated at 10% *v*/*v* in order to determine the more adequate carbon source concentration to produce BC at eight days, following the proposed parameters by Castro et al. [32]. The response variable for this analysis was the amount of cellulose produced (g/L) and BC produced in the medium with the more adequate carbon source concentration was selected to be characterized. The experiments were performed in triplicate.

### 4.2. Biomass Quantification 

Microorganism biomass was measured by optical density, and 3 mL of the samples were taken every 10 h, 3.3% *v*/*v* cellulase (Celuclast-Novozymes) was added to the sample and incubated at 50 °C in a water bath for two hours to degrade the cellulose and take readings of absorbance at 600 nm [48]. This procedure was repeated every 10 h to a total time of 100 h to use these data to building the curves for growth of *K. medellinensis* in each culture media.

### 4.3. Consumption Dynamics of Carbon Sources

The analysis of carbon source consumption was performed using a HPLC. Before the measurement, culture media samples were filtered through a polyethersulfone (PES) filter of 0.450 μm. Samples were injected onto ION exclusion column of 300 mm × 7.800 mm with a solution of sulfuric acid (0.005 N) at a flow rate of 0.600 mL/min at 30 °C. For nitrogen source consumption quantification, the Biuret colorimetric test was used, for which 1 L of standard Biuret solution and 10 mL of the standard solution BSA was prepared.

### 4.4. Bacterial Cellulose Characterization

The extracted membranes after 15 days of fermentation were washed with KOH 5% (*w*/*v*) aqueous solution during 14 h at 28 °C to remove medium components and bacteria attached, then washed with water to neutral pH.

### 4.5. Scanning Electronic Microscopy (SEM)

SEM was used to observe the nanoribbon network morphology and its distribution in the membrane. The membranes were cut, freeze-dried, and coated with gold/palladium using an ion sputter coater. Samples were observed with a Jeol JSM 5910 LV microscope (Jeol, Tokyo, Japan) operated at 20 kV and the images were analyzed by Image-J software for BC porosity determination.

### 4.6. Attenuated Total Reflection Fourier Transformed Infrared Spectroscopy

Before the measurement, the cellulose membranes were dried for 2 h at 100 °C to remove moisture. ATR-FT-IR spectra were recorded on a Nicolet 6700 spectrophotometer (Thermo Scientific, Waltham, MA, USA) in the 4000–400 cm^−1^ range ATR with a diamond crystal. The spectra were recorded with a resolution of 4 cm^−1^ and an accumulation of 256 scans.

The ratio of cellulose I_α_ and I_β_ was measured according to Imai and Sugiyama, using the integrated intensities of the two-absorbance 710 cm^−1^ and 750 cm^−1^ peaks trough the relationship described in Equation (1):

(1) $$f_{\propto }\;=\;\frac{A_{750}}{A_{750}\;+\;{\mathrm{kA}}_{710}}$$

where *A_750_* is the absorbance of 750 cm^−1^ band, *A_710_* is the absorbance of 710 cm^−1^ band, and *k* is the ratio of adsorption coefficients between those bands [48].

### 4.7. X-ray Diffraction

Dried cellulose membranes with different thicknesses were analyzed by X-ray diffraction on a Bruker model D8 Advance (Bruker, Billerica, MA, USA) with DaVince geometry equipment operating at the Ni-filtered CuKα_1_ radiation wavelength (λ = 0.15406 nm), generated at a voltage of 45 kV and a filament emission of 40 mA. Data were collected in reflection mode in the 10–30° 2θ-range with a step of 0.013°. The scans proceeded at 56.58 s per step. The d-spacings between the crystal planes were determined using Bragg’s law expressed by Equation (2):

(2) $$d=\frac{\lambda }{2\sin\theta }$$

where θ is the angle between the plane and the diffracted or incident beam and λ is the wavelength of the X-rays. An apparent crystal size (*ACS*) approximation was calculated using Scherrer’s formula (Equation (3)):

(3) $$\mathrm{ACS}=\frac{0.9\lambda }{\mathrm{FWHM}\cos\theta }$$

where FWHM is the width of the peak at half the maximum height, θ is Bragg’s angle, and λ is the wavelength of the X-rays.

Crystallinity index (CI) was calculated according to second method described by Park et al., as the ratio of the area of all crystalline peaks to the total area.

## 5. Conclusions

This study considers the metabolic requirements of a new microorganism, namely, pH-resistant *Komagataeibacter medellinensis*. The effect of different carbon sources and their concentration in the production and structure of bacterial nanocellulose (BC) was studied. The highest BC yield was found after eight days of fermentation in 2% *w*/*v* of glucose (2.80 g/L), followed by sucrose (1.68 g/L) and fructose (0.38 g/L). After 15 fermentation days, the BC yield was the same in glucose and sucrose (3.3 g/L). The results indicate the presence of fructose in the sucrose medium, which could trigger the exopolysaccharide (EPS) production (as cellulose) as a defense mechanism against osmotic stress. Despite this, sucrose hydrolysis was produced by the action of citric acid and temperature during the sterilization process and not by *K. medellinensis*, as has been suggested by other authors. The biomass production was affected by carbon source, obtaining the highest production in the glucose medium and the lowest in the fructose medium, which affected the morphological characteristics of the produced BC. In fact, a reduced BC porosity was noted in the fructose medium while a higher crystallinity index was determined. The chemical features of BC were not affected by the carbon source. Our results are relevant to any attempt to produce BC, to adjust the production conditions and to understand how BC could be affected by changes in culture media (carbon source and concentration).

## Figures and Tables

**Table 1:** Product-substrate yield (Y_PS_) for each culture medium evaluated. Y_PS_ was calculated as the ratio of dry cellulose produced and substrate consumed during fermentation.

Carbon Source	Product-Substrate Yield (g/g)
Fructose	0.20
Glucose	0.19
Sucrose	0.59

**Table 2:** Porosity of BC produced from different culture media.

Carbon Source	Porosity (%)
Fructose	45.23 (±1.92)
Glucose	60.07 (±0.55)
Sucrose	54.70 (±1.47)

**Table 3:** I_α_ fraction estimation in each BC produced from the evaluated culture media.

Substrate	Allomorph I_α_ Fraction Estimation (A_750_/A_710_)
Fructose	0.70 (±0.02)
Glucose	0.74 (±0.05)
Sucrose	0.71 (±0.00)

**Table 4:** D-spacing, ACS, and crystallinity index in different media.

Substrate	100		010		110		CI
	D-Spacing (nm)	ACS (nm)	D-Spacing (nm)	ACS (nm)	D-Spacing (nm)	ACS (nm)	
Fructose	0.60	8.83	0.51	8.07	0.39	10.66	0.90 (±0.02)
Glucose	0.60	8.66	0.52	8.33	0.39	10.52	0.83 (±0.03)
Sucrose	0.60	8.43	0.52	8.05	0.38	10.65	0.85 (±0.06)
